# CTLA-4 polymorphisms and haplotype correlate with survival in ALL after allogeneic stem cell transplantation from related HLA-haplotype-mismatched donor

**DOI:** 10.1186/s12967-016-0864-2

**Published:** 2016-04-27

**Authors:** X.-Y. Qin, Y. Wang, G.-X. Li, Y.-Z. Qin, F.-R. Wang, L.-P. Xu, H. Chen, W. Han, J.-Z. Wang, X.-H. Zhang, Y.-J. Chang, K.-Y. Liu, Z.-F. Jiang, X.-J. Huang

**Affiliations:** Peking University People’s Hospital, Peking University Institute of Hematology, Beijing, China; Beijing Key Laboratory of Hematopoietic Stem Cell Transplantation, Beijing, China; Peking-Tsinghua Center for Life Sciences, Beijing, China; State Key Laboratory of Protein and Plant Gene Research, Key Laboratory of Cell Proliferation and Differentiation of the Ministry of Education school of Life Sciences, Peking University, Beijing, China; Peking University-Tsinghua University Joint Center for Life Sciences, Beijing, China; Peking University People’s Hospital, Peking University Institute of Hematology, Beijing Key Laboratory of Hematopoietic Stem Cell Transplantation, Peking-Tsinghua Center for Life Sciences, 11 Xizhimen South Street, Beijing, 100044 Peoples’ Republic of China

**Keywords:** Allogeneic stem cell transplantation, Haplotype, CTLA-4 polymorphisms, Overall survival, ALL

## Abstract

**Background:**

Allogeneic hematopoietic stem cell transplantation (allo-HSCT) has been established as an effective treatment for patients with hematological malignancies. Disease relapse remains a major cause of transplant failure. T cell homeostasis is critical to determine the potency of the GVT effect. Recent studies have shown the association of the CTLA-4 polymorphisms with the outcome after HLA-identical sibling allogeneic HSCT.

**Methods:**

In this study, we focused on four CTLA-4 polymorphisms, and analyzed the impact of donor genotypes and haplotypes on the conditions of 152 acute leukemia patients (ALL 83) after related HLA-haplotype- mismatched transplantation. The four SNP genotypes (−1661, −318, CT60 and +49) were determined by TaqMan SNP genotyping assays.

**Results:**

ALL recipients of donors with +49 GG showed significantly lower OS (67.7 vs. 90.3 %, P = 0.015) than those with GA+AA. Multivariate analyses showed that +49 GG was an independent risk factor for OS (HR: 0.306, 95 % CI 0.111–0.842, P = 0.022) .23 ALL patients receiving mDLI showed significantly lower OS with +49 GG donor than those with GA+AA (30.0 vs. 83.1 %, P = 0.003). The haplotype analysis revealed only three haplotypes in the donor population −1661/−318/CT60/+49 i.e., ACGG, ACAA and GTGA, the frequencies were 64.1, 19.4 and 16.5 %, respectively. Donors with and without the ACGG/ACGG haplotype had the same effect on transplant outcomes as those with +49 GG and +49 GA+AA.

**Conclusion:**

In summary, the CTLA-4 +49 GG and the haplotype ACGG/ACGG reduced the overall survival in ALL after allo-HSCT from the related HLA-haplotype-mismatched donor, knowledge of the CTLA-4 polymorphism and haplotype may provide useful information for donor selection and individual application of immunosuppressive agents and immunotherapy.

## Background

Allogeneic hematopoietic stem cell transplantation (allo-HSCT) has been established as an effective treatment for patients with hematological malignancies. Disease relapse remains a major cause of transplant failure. Although several clinical variables including disease status at transplant, stem cell source, graft-versus-host disease (GVHD) prophylaxis, and conditioning regimen contribute to the risk of relapse, the predictors of graft-versus-tumor (GVT) effect remain largely elusive. T cell homeostasis is critical to determine the potency of the GVT effect [1–3].

Cytotoxic T lymphocyte antigen-4 (CTLA-4 or CD152) is a member of the immunoglobulin superfamily and is a T cell activation negative regulator. After the initial recognition of the antigen by the T cell receptor complex, the primary positive co-stimulatory signal is the T cell CD28, which binds with CD80/CD86 on antigen-presenting cells. CTLA-4 is expressed in T cells after activation and exerts its negative regulatory effects by competing with CD28 for CD80/CD86 and consequently blocking downstream activation pathways through transendocytosis and degradation. CTLA-4 is highly conserved among mammals, thereby indicating the importance of inhibiting proliferation and inducing peripheral tolerance in T cells [4]. CTLA-4 is a receptor expressed on the activated T cell surface and is a homolog of CD28 that is responsible for T cell activation. Although both CTLA-4 and CD28 bind with the two ligands B7.1 (CD80) and B7.2 (CD86) expressed on APCs, CTLA-4 binds B7 molecules with higher affinity and avidity than CD28. CTLA-4 gene polymorphisms correlate with autoimmune diseases such as systemic lupus erythematosus [5, 6], type 1 diabetes mellitus [7, 8] and Graves’ disease [9]. Recent studies have shown the association of the CTLA-4 polymorphisms [−1661 (rs4553808), −318 (rs5742909), +49 (rs231775), and CT60 (rs3087243)] with the outcome after HLA-identical sibling allogeneic HSCT [10–12]. We reported our approach to related HLA-haplotype-mismatched T cell-replete transplants in >1000 subjects in the last 10 years [13, 14]. In the present study, we focused on these four CTLA-4 polymorphisms and analyzed the impact of donor genotypes and haplotypes in 152 acute leukemia (ALL 83) patients on the outcomes after related HLA-haplotype-mismatched transplantation.

## Patients and methods

### Patients

Between January 2010 and December 2012, a total of 152 consecutive AL patients who underwent allo-HSCT at the Peking University Institute of Hematology and the survivors until day 100 of post-allo-HSCT were included in this study. All patients received related HLA-haplotype-mismatched T-cell-replete transplants. Each subject received a graft from a family member sharing one HLA haplotype with the recipient but differed to a variable degree for the HLA-A, B, and DR antigens of the unshared HLA haplotype. All patients provided informed consent for treatment under a protocol reviewed and approved by the Peking University Institute of Hematology. The characteristics of the patients and transplantations are summarized in Table 1.

**Table 1:** Patients chracteristics and outcomes

Characteristics	No. of patients (%)	No. of relapse (%)
Number of patients	152	34 (22.4)
Median age, years (range)	23 (2–55)	18.5 (2–55)
Sex (male/female)	85/67	19/15
Disease type		
ALL	83 (54.6)	23 (67.6)
AML	69 (45.4)	11 (32.4)
Disease status before HSCT		
SR	117 (77.0)	29 (85.3)
HR	35 (23.0)	5 (14.7)
MRD status before HSCT		
MRD− remission	104 (68.4)	20 (58.8)
MRD+ remission	43 (28.3)	12 (35.3)
Refractory disease	5 (3.3)	2 (5.9)
HLA-haploidentical related donor (the number of mismatched locus)		
3	93 (61.2)	19 (55.9)
2	49 (32.2)	12 (35.3)
1	10 (6.6)	3 (8.8)
Donor-recipient blood type		
Match	84 (55.2)	19 (55.9)
Minor mismatch	29 (19.1)	4 (11.8)
Major mismatch	27 (17.8)	9 (26.4)
Minor + major	12 (7.9)	2 (5.9)
aGVHD	48 (31.6)	12 (35.3)
>aGVHD II	8 (5.3)	2 (5.9)
cGVHD	81 (53.3)	14 (41.2)
Extensive cGVHD	27 (17.8)	5 (14.7)
Median follow-up time (days)	633.5 (112–1369)	385.5 (112–1369)
Death (%)	31 (20.4)	19 (55.9)
Transplantation related death (%)	12 (7.9)	
IFD	13 (8.6)	2 (5.9)
CMV infection	117 (77.0)	25 (73.5)

*AML* acute myeloid leukemia, *ALL* acute lymphoblastic leukemia, *SR* standard risk, *HR* high-risk, *MRD* minimal residual disease, *IFD* invasive fungal disease, *CMV* cytomegalovirus

### Transplant protocols

All patients received myeloablative conditioning regimens [15–17]. Busulfan/cyclophosphamide (BU/CY)-based conditioning therapy was administered (4 mg/kg/d BU orally for 3 days, and 1.8 g/m^2^/day CY i.v. for 2 days). A single dose of semustine (250 mg/m^2^) was orally administered to all human leukocyte antigen (HLA)-mismatched and related patients. They were also given cytarabine (4 g/m^2^/day) and anti-thymocyte globulin (2.5 mg/kg/day, SangStat, Lyon, France) i.v. for 2 and 4 consecutive days, respectively. All subjects received granulocyte colony-stimulating factor (G-CSF)–mobilized BM and blood cells. Prophylaxis against GVHD involved the treatment with cyclosporine A (CsA) and short-term methotrexate (MTX) with mycophenolate mofetil. On day 9 pre-HSCT, CsA (2.5 mg/kg) was treated i.v., and mycophenolate mofetil was administered orally (0.5 g/12 h) from day 9 before the transplantation until day 30 after the transplantation. At 30 day after the transplantation, 0.25 g of mycophenolate mofetil was administered every 12 h for 1–2 months. On day 1, MTX (15 mg/m^2^) was administered i.v. and 10 mg/m^2^ MTX was given on days 3, 6, and 11 after transplantation. Immunosuppression regimens were discontinued for all patients who showed AL relapse or increased recipient chimerism while still taking immunosuppressive agents.

### Sample preparation and CTLA-4 genotyping

Donor BM or PB samples from 152 allo-HSCT patients were obtained to investigate CTLA4 polymorphisms after transplantation. DNA molecules from donor BM and PB samples were extracted using the Qiagen Midi Kit and Qiagen Mini kit (Qiagen, Hilden, Germany). The concentration and purity of each DNA sample were evaluated by measuring the optical density at 260 and 280 nm in a UV spectrometer. The four SNP genotypes of CTLA-4, namely, −1661 (rs4553808), −318 (rs5742909), CT60 (rs3087243), and +49 (rs231775), were determined by TaqMan SNP genotyping assays (Applied Biosystems) according to manufacturer instructions. PCR was performed in a 10 μL reaction volume containing 5 μL TaqMan Universal Master Mix (Applied Biosystems, Foster City, CA, USA), 1 mmol of each primer, 1 mmol of each probe, and 1 mmol of genomic DNA. Thermal cycle conditions were 95 °C for 10 min, 40 cycles of 95 °C for 15 s, and 60 °C for 1 min. All PCR and endpoint fluorescent readings were analyzed using an ABI 7300 Real-Time PCR System (Applied Biosystems).

### Minimal residual disease (MRD) monitoring and definition

The MRD level was determined via real-time quantitative PCR and four-color flow cytometry (FCM) on bone marrow aspirates after HSCT [18, 19]. The MRD markers included leukemia-associated immune phenotypes (LAIP) and leukemia-associated genes (WT1, AML1-ETO, CBFB-MYH11, and BCR-ABL) assayed in diagnostic specimens. More than 0.001 % of LAIP via four-color FCM or more than 10^−4^ of reciprocal fusion genes or 0.6 % of WT1 gene via real-time quantitative PCR in BM samples was considered as abnormal. The definition of MRD was two consecutive abnormalities for FCM or leukemia-associated genes during a 15–30 days interval.

### Modified donor lymphocyte infusion (mDLI)

After diagnosing the MRD and the increasing recipient chimerism [20], pre-emptive mDLI was performed to prevent relapse after HSCT. Post-transplantation immune suppression was immediately tapered and subsequently discontinued in patients who were in less than 100 days posttransplant. Patients who were in more than 100 days posttransplant had the immune suppression immediately discontinued. In patients without active GVHD, mDLI was given based on the DLI donor availability. Anti-leukemia chemotherapy were administered to patients 48–72 h before mDLI; this therapy consisted of 20 mg aclacinomycin and 150 mg/m^2^ cytarabine (both administered for 7 days) in acute myeloid leukemia (AML) patients and 1 g/m^2^ MTX (for 1 day) in acute lymphoblastic leukemia (ALL) patients. For mDLI, G-CSF–mobilized peripheral blood stem cells were administered instead of the more common, unstimulated, donor blood lymphocytes. After mDLI, patients received immunosuppressive drugs such as CsA or MTX to prevent GVHD. Patients who received mDLI from an HLA-haploidentical donor received GVHD prophylaxis for 4–6 weeks at the discretion of the attending physicians and depending on the GVHD status of the patient after mDLI. The starting dose of CsA was 2.5 mg/kg/day and was adjusted to maintain a plasma concentration of >100 ng/mL. Moreover, 10 mg MTX was administered i.v. on days 1, 4, and 8 and weekly thereafter for 2–6 weeks [15, 16]. Chimerism and MRD status were monitored at 1, 2, 3, 4.5, 6, 9 and 12 months after the interventions, and at 6-month intervals thereafter.

### Study definitions

Neutrophil engraftment had an absolute neutrophil count (ANC) of >0.5 × 10^9^/L for three consecutive days. Platelet engraftment without transfusion had platelet count of >20 × 10^9^/L for seven consecutive days. The diagnosis and grading of GVHD was established based on published criteria [15–17]. Patients with malignancies in first or second complete remission (CR1 or CR2, respectively) of AL were categorized as “standard risk.” Patients in more than the second complete remission of AL, not in remission, or had high-risk cytogenetics, such as t(9;22) and t(4;11), were classified as “high risk.” Moreover, complete remission (CR) was defined as BM blasts <5 %, absence of blasts with Auer rods, absence of extramedullary disease, ANC of >1.0 × 10^9^/L, platelet count of >100 × 10^9^/L, and independence of red cell transfusions. Partial remission (PR) included all hematological criteria of CR, BM blast percentages from 5 to 20 %, and a decrease of pretreatment BM blast percentage by a minimum of 50 %. Patients who did not achieve either of the mentioned standards (for CR or PR) were classified as no remission (NR). Relapse was defined as recurrence of BM blasts >5 %, reappearance of blasts in the blood, or development of extramedullary disease infiltrates at any site whereas relapse rate was the probability of leukemia recurrence. Over survival (OS) was calculated from the date of transplantation until death or last observation of patients’ life. Disease-free survival (DFS) was described as the probability of being alive and free of disease at any point in time. MRD targets were also regularly examined within 2 weeks before transplantation. The MRD status before HSCT was defined as abnormal if it contained more than 0 0.001 % of LAIP via four-color FCM, more than 10^−4^ of reciprocal fusion genes, or more than 0.6 % of Wilms tumor gene 1 (WT1) [18, 19, 21]. Refractory disease included NR and PR patients before HSCT. A diagnosis of proven or probable invasive fungal disease (IFD)was determined by the revised European Organization for Research and Treatment of Cancer and Mycoses Study Group (EORTC/MSG) criteria [22], only proven and probable cases were included in the determination of the cumulative incidence of IFD. Cytomegalovirus (CMV) infection was defined as isolation of CMV virus or detection of viral proteins or nucleic acid in any body fluid or tissue specimen.

### Statistical analysis

The reference date of 1 October 2013 was used to define the end of the follow-up period. The distribution of genotypes in the cases were calculated with Hardy–Weinberg equilibrium test. The statuses of DFS and OS were calculated according to the Kaplan–Meier statistics. The differences in DFS and OS between groups were calculated using the log-rank test. A two-sided P value of 0.05 was regarded as significant. The multivariate Cox regression analysis was applied to examine the independence of predictive factors. Variables considered in the multivariate models were donor sex (male vs. female), donor age (continuous variable), HLA mismatching locus, Donor-recipient blood type, status of disease before HSCT, MRD status before HSCT, IFD and CMV infection. CMV serological state before HSCT was not considered as a covariate, because only 1 % of subjects were low risk (recipient-, donor-) for CMV reactivity. The independence of categorical parameters was calculated using the χ^2^-test or Fisher’s exact test, whereas the distribution of continuous variables was determined using the Mann–Whitney U-test.

## Results

### General patient information

All patients achieved neutrophil engraftment at a mean time of 12 day (10–25 day). Seven patients achieved platelet engraftment after 100 day, and one died at 5 months before the platelet engraftment. The median time of platelet engraftment was 15 day (6–225 day). By the 1 October 2013 end point, the median follow-up time was 633.5 day (112–1369 day). A total of 48 patients (31.6 %) developed acute GVHD, and 8 (5.3 %) developed acute GVHD higher than the grade II. Chronic GVHD developed in 81 patients (53.3 %), and extensive chronic GVHD developed in 27 patients (17.8 %). Overall, 34 patients (22.4 %) relapsed after transplantation (median 172 day, 32–1076 day). A total of 31 patients (20.4 %) died because of relapse (n = 19) or other causes of transplantation-related mortality (n = 12; infection, n = 9; cerebral hemorrhage, n = 1; hemorrhage of digestive tract, n = 1; and post-transplantation lympho-proliferative disorders, n = 1) (Table 1).

### Frequencies of CTLA-4 genotypes and haplotypes

The frequencies at which the four CTLA-4 SNPs were expressed in the 154 donors are listed in Table 2. The SNP −1661 allele A was blocked by the SNP −318 allele C. The SNP −1661, SNP −318, CT60, and +49 were included in one haplotype block that was constructed using the international HapMap database. The haplotype analysis revealed only three haplotypes in the donor population: −1661*A/−318*C/CT60*G/+49*G (ACGG), −1661*A/−318*C/CT60*A/+49*A (ACAA), and −1661*G/−318*T/CT60*G/+49*A/(GTGA). In this cohort, the frequencies of the haplotype ACGG, ACAA and GTGA were 64.1, 19.4 and 16.5 %, respectively. All of the donors were distributed among the six haplotype combinations (Table 3).

**Table 2:** Allele and genotype frequencies at the CLTA-4 loci in 152 donors

Polymorphism	All donors (%)	Donors in ALL (%)	Donors in AML (%)
No of donors	152	83	69
−1661 (rs4553808)			
AA	109 (71.7)	61 (73.5)	48 (69.6)
AG	36 (23.7)	18 (21.7)	18 (26.1)
GG	7 (4.6)	4 (4.8)	3 (4.3)
A allele	254 (83.6)	140 (84.3)	114 (82.6)
G allele	50 (16.4)	26 (15.7)	24 (17.4)
HWE P^a^	0.09	0.10	0.44
−318 (rs5742909)			
CC	109 (71.7)	61 (73.5)	48 (69.6)
TC	36 (23.7)	18 (21.7)	18 (26.1)
TT	7 (4.6)	4 (4.8)	3 (4.3)
C allele	254 (83.6)	140 (84.3)	114 (82.6)
T allele	50 (16.4)	26 (15.7)	24 (17.4)
HWE P^a^	0.09	0.10	0.44
CT60 (rs3087243)			
GG	96 (63.2)	51 (61.5)	45 (65.2)
AG	53 (34.9)	30 (36.1)	23 (33.3)
AA	3 (1.9)	2 (2.4)	1 (1.5)
G allele	245 (80.6)	132 (79.5)	113 (81.9)
A allele	59 (19.4)	34 (20.5)	25 (18.1)
HWE P^a^	0.16	0.32	0.30
+49 (rs231775)			
GG	59 (38.8)	31 (37.3)	28 (40.6)
AG	77 (50.7)	44 (53.0)	33 (47.8)
AA	16 (10.5)	8 (9.7)	8 (11.6)
G allele	195 (64.1)	106 (63.9)	89 (64.5)
A allele	109 (35.9)	60 (36.1)	49 (35.5)
HWE P^a^	0.21	0.18	0.71

^a^The distribution of genotypes was consistent with Hardy–Weinberg equilibrium (P > 0.05)

**Table 3:** Frequencies of the CTLA-4 haplotype in 152 donors

haplotype	−1661	−318	CT60	+49	All 152 donors (%)	Donors in ALL (%)	Donors in AML (%)
A-C-G-G/A-C-G-G	AA	CC	GG	GG	59 (38.8)	31 (37.4)	28 (40.6)
A-C-G-G/A-C-A-A	AA	CC	GA	GA	47 (30.9)	28 (33.7)	19 (27.5)
A-C-G-G/G-T-G-A	AG	CT	GG	GA	30 (19.7)	16 (19.3)	14 (20.3)
A-C-A-A/A-C-A-A	AA	CC	AA	AA	3 (2.0)	2 (2.4)	1 (1.5)
A-C-A-A/G-T-G-A	AG	CT	AG	AA	6 (4.0)	2 (2.4)	4 (5.8)
G-T-G-A/G-T-G-A	GG	TT	GG	AA	7 (4.6)	4 (4.8)	3 (4.3)
A-C-G-G					195 (64.1)	106 (63.9)	89 (64.5)
A-C-A-A					59 (19.4)	34 (20.4)	25 (18.1)
G-T-G-A					50 (16.5)	26 (15.7)	24 (17.4)

### Effect of the four CTLA-4 SNPs on transplant outcome

**Fig. 1:**
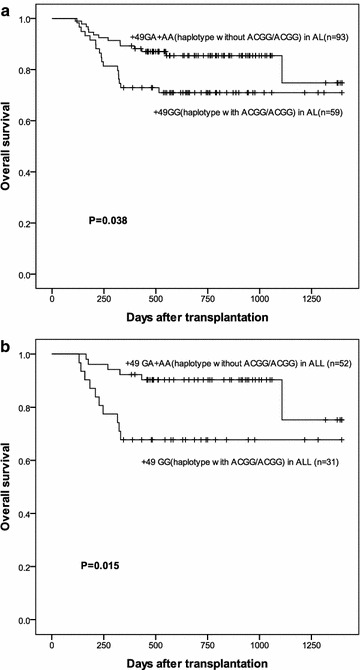
The effect of +49 genotype and haplotype on OS in AL and ALL patients. **a** OS in AL patients according to +49 genotype(GG/GA+AA) and haplotype with or without ACGG/ACGG (P = 0.038). **b** OS in ALL patients according to +49 genotype (GG/GA+AA) and haplotype with or without ACGG/ACGG (P = 0.015)

The Kaplan–Meier analysis revealed that recipients of donors with +49 GG showed a significantly lower OS (71.0 vs. 85.5 %, P = 0.038) than those with GA+AA (Fig. 1a). No significant effect was found on OS of SNP −1661 AA/GA+GG (P = 0.208), SNP −318 CC/CT+TT (P = 0.208), and CT60 GG/GA+AA (P = 0.169). Moreover, the recipients showed no significant difference in cumulative incidence of relapse (CIR) and DFS in four CTLA-4 SNPs.

The ALL recipients of donors with +49 GG showed significantly lower OS (67.7 vs. 90.3 %, P = 0.015) than those with +49 GA+AA (Fig. 1b). No significant difference was observed in the OS between the AML recipients of donors with +49 GG and with GA+AA (P = 0.67). Univariate analyses showed that treatment with the +49 GG was a risk factor for OS in ALL (HR: 0.306, 95 % CI 0.111–0.842, P = 0.022) (Table 4). Cox multivariate analysis considered several variables including the age, sex, HLA mismatching locus, status of disease before HSCT, MRD status before HSCT, donor-recipient blood type,IFD,CMV infection. Multivariate analyses showed that the presence of +49 GG was an independent risk factor for OS in ALL (HR: 0.306, 95 % CI 0.111–0.842, P = 0.022).

**Table 4:** Univariate analyses of the CTLA-4 SNP and haplotype on outcomes in ALL after HSCT

	−1661 AA/AG+GG	−318 CC/CT+TT	CT60 GG/GA+AA	+49 GG/GA+AA	With ACGG/ACGG	Without ACAA
Relapse						
P	0.779	0.779	0.738	0.382	0.382	0.738
HR	0.875	0.875	0.864	0.692	0.692	0.864
95 % CI	0.344–2.223	0.344–2.223	0.366–2.041	0.303–1.580	0.303–1.580	0.366–2.041
DFS						
P	0.575	0.575	0.666	0.240	0.240	0.666
HR	0.784	0.784	0.844	0.645	0.645	0.844
95 % CI	0.336–1.837	0.336–1.837	0.392–1.818	0.310–1.341	0.310–1.341	0.392–1.818
OS						
P	0.373	0.373	0.101	0.022^#^	0.022^#^	0.101
HR	0.565	0.565	0.350	0.306	0.306	0.350
95 % CI	0.161–1.984	0.161–1.984	0.100–1.229	0.111–0.842	0.111–0.842	0.100–1.229
aGVHD III-IV						
P	0.527	0.527	0.862	0.324	0.324	0.862
HR	0.030	0.030	0.809	0.299	0.299	0.809
95 % CI	0.000–1534.4	0.000–1534.4	0.073–8.917	0.027–3.294	0.027–3.294	0.073–8.917
Extensive cGVHD						
P	0.516	0.516	0.045^#^	0.273	0.273	0.045^#^
HR	0.655	0.655	3.067	2.041	2.041	3.067
95 % CI	0.183–2.350	0.183–2.350	1.027-9.159	0.569–7.322	0.569–7.322	1.027–9.159

*HR* indicates hazard ratio, *CI* confidence interval, *HSCT* hematopoietic stem cell transplantation

^#^P < 0.05

**Fig. 2:**
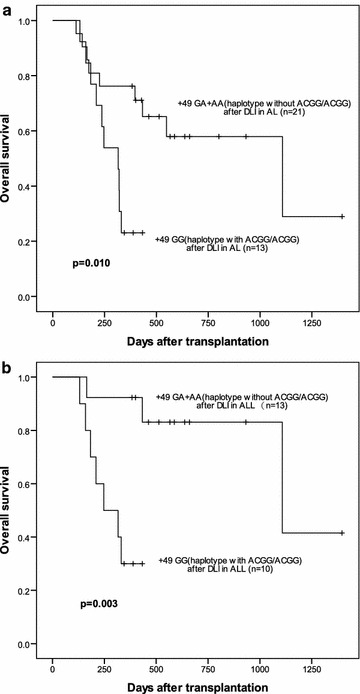
The effect of +49 genotype and haplotype on OS in AL and ALL patients after DLI. **a** OS after DLI in AL patients according to +49 genotype(GG/GA+AA) and haplotype with or without ACGG/ACGG (P = 0.010). **b** OS after DLI in ALL patients according to +49 genotype(GG/GA+AA) and haplotype with or without ACGG/ACGG (P = 0.003)

A total of 34 (22.4 %) patients relapsed after related HLA-haplotype-mismatched transplantation, all of these patients received modified DLI. The Kaplan–Meier analysis indicated a significantly lower OS in patients receiving mDLI with +49 GG donor than those with GA+AA (23.1 vs. 57.9 %, P = 0.010) (Fig. 2a). Further analyses showed a significantly lower OS in ALL patients receiving mDLI with the +49 GG donor than those with GA+AA (30.0 vs. 83.1 %, P = 0.003) (Fig. 2b). No significant difference was also observed between AML patients receiving mDLI with the +49 GG donor and with GA+AA (P = 0.535).

**Fig. 3:**
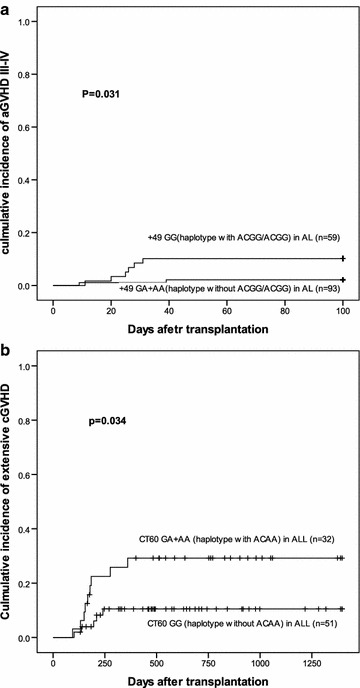
The effect of +49 and CT60 genotype and haplotype on GVHD in AL and ALL patients. **a** aGVHD III-IV in AL patients according to +49 genotype(GG/GA+AA) and haplotype with or without ACGG/ACGG (P = 0.031). **b** Extensive cGVHD in ALL patients according to CT60 genotype (GG/GA+AA) and haplotype without or with ACAA (P = 0.034)

Recipients of donors with +49 GG showed significantly higher incidence of aGVHD III–IV (10.2 vs. 2.2 %, P = 0.031) than those with GA+AA (Fig. 3a). However, no significant differences in aGVHD II–IV, cGVHD, and extensive cGVHD were observed between recipients of donors with the +49GG and GA+AA (P = 0.927, P = 0.478, P = 0.956). Univariate analyses and multivariate analyses did not reveal that the +49 GG was a risk factor for aGVHD III–IV (HR: 0.204, 95 % CI 0.041–1.010, P = 0.051). Recipients of donors with CT60 GG in ALL showed significantly lower incidence of extensive cGVHD (10.5 vs. 29.2 %, P = 0.034) than those with GA+AA (Fig. 3b), Univariate analyses and multivariate analyses reveal that the CT60 GG was a risk factor for extensive cGVHD (HR: 3.067, 95 % CI 1.027–9.159, P = 0.045). Moreover, no significant difference in aGVHD III–IV, aGVHD II–IV, cGVHD, and extensive cGVHD in the SNP −1661, SNP −318 was observed.

There were no significant association of CTLA-4 polymorphisms with risk of infection after transplantation. No significant difference was observed in the IFD between the donors with +49 GG and with GA+AA (P = 0.534). No significant difference was observed in the CMV infection between the donors with +49 GG and with GA+AA (P = 0.870).

### Effect of the CTLA-4 haplotype on transplant outcome

SNP −1661, SNP −318, CT60, and +49 were included in one haplotype block that was constructed using the international HapMap database. The haplotype analysis revealed only three haplotypes in the donor population, as follows: −1661*A/−318*C/CT60*G/+49*G (ACGG), −1661*A/−318*C/CT60*A/+49*A (ACAA) and −1661*G/−318*T/CT60*G/+49*A (GTGA). We therefore focused our analysis on the ACGG and ACAA haplotypes and examined their association with the outcome after allo-HSCT. Donors with and without the ACGG/ACGG haplotype have the same effect on transplant outcome as that of donors with the +49 GG and +49 GA+AA. Donors without and with the ACAA haplotype have the same effect on transplant outcome as that of donors with the CT60 GG and CT60 GA+AA.

## Discussion

In this study, we focused on the four CTLA-4 SNP polymorphisms and analyzed the impact of donor genotypes and haplotypes on the outcome in AL patients after related HLA-haplotype-mismatched transplantation. Although the association of CTLA-4 SNP and the outcome after allo-HSCT have been previously reported [10–12], we are the first to report the relationship between CTLA-4 SNP and the transplantation outcome in AL patients from related HLA-haplotype-mismatched donor.

The human CTLA-4 gene is located on 2q33. The CTLA-4 gene is composed of four exons and has two isoforms, a full-length isoform (flCTLA-4) and a soluble form (sCTLA-4) that lacks exon 3 that encodes the transmembrane domain. flCTLA-4 down-regulates T cell responses by inducing cell cycle arrest and blocking cytokine production [23, 24]. Many studies have reported increased sCTLA-4 protein levels in patients with autoimmune disease [25–28]. Serum sCTLA-4 has the potential to bind to CD80/CD86 [29, 30]; sCTLA-4 blocks the interaction of flCTLA-4 with CD80/CD86 and thereby enhances T-cell activation. CT60 maps at the 3′ untranslated region of CTLA-4 could play a role in the alternative splicing by which sCTLA-4 is generated. The CT60 A allele has been reported to be associated with a higher level of the sCTLA-4 mRNA than the CT60 G allele [10, 31]. The SNP −318 is located at the CTLA-4 promoter region. Previous studies showed that the −318 C allele is correlated with a lower promoter activity and a lower CTLA-4 expression than those observed with the −318 T allele [32, 33]. The SNP −1661 is in the upstream promoter region of CTLA-4. Although the −1661 is less characterized than other SNPs in the CTLA-4 gene [34], it has been associated with increased risk of multiple sclerosis [35], type 1 diabetes mellitus [36, 37] systemic sclerosis [38], oral squamous cell carcinoma [39], and lymph node involvement in breast cancer [40]. Testing the recombinant proteins of the various CTLA-4 variations, or isolating T cells from the patients with benign versus malignant CTLA-4 SNPs to test overall CTLA-4-mediated immunosuppression, can provide further evidence to test the impact of this SNP and haplotype. Further research should be carried out in this field in the future.

In our study, the CTLA-4 +49 GG and the haplotype ACGG/ACGG reduced the overall survival in ALL after allo-HSCT from the related HLA-haplotype-mismatched donor (67.7 vs. 90.3 %, P = 0.015). The probable mechanisms that ALL patients’outcomes might be influenced by CTLA-4 polymorphisms were as followed: The CTLA-4 exon 1, where the +49 polymorphism is located, encodes the leader peptide of the protein, which is responsible for CTLA-4 trafficking to the endoplasmic reticulum. Several previous studies reported that the CTLA-4 +49 polymorphism alters the inhibitory effect of CTLA-4 on T-cell activation [31, 41–43]. CTLA-4 proteins contribute to the suppressor function of regulatory T cells (Tregs), a protein that is present in Tregs and the expression of which in Tregs is dependent on Foxp3 is CTLA-4 [44]. Mice with Tregs that lack CTLA-4 protein expression were shown recently to develop lethal autoimmunity, revealing that Treg expression of CTLA-4 was necessary for immune suppression and prevention of in vivo autoimmunity [45, 46]. Ipilimumab, a CTLA-4 antibody, has been studied in melanoma. Recently, Hodi et al. [47] have shown that ipilimumab improves OS in patients with melanoma. In patients with melanoma, the G allele of rs4553808 in CTLA-4 was associated with improved response to ipilimumab [48]. In a phase I clinical trial of ipilimumab after allo-HSCT, complete remission was achieved in two patients with Hodgkin’s lymphoma and partial remission in one patient with refractory mantle cell lymphoma, suggesting that targeting CTLA-4 is feasible [49]. The mechanism of why ALL patients’ outcomes might be influenced by CTLA-4 polymorphisms and haplotype needs further investigated. Recently PD-L1, PD-L2, LAG-3, IDO1 etc. have been reported to correlate to immune defects [50],to study the relation of CTLA4, PD-L1, PD-L2, LAG-3, IDO1, etc. with the evolution of immune response of HSCT patients will be needed in the future.

Recipients of donors with +49 GG showed significantly higher incidence of aGVHD III–IV (10.2 vs. 2.2 %, P = 0.031) than those with GA+AA. Recipients of donors with CT60 GG in ALL showed significantly lower incidence of extensive cGVHD (10.5 vs. 29.2 %, P = 0.034) than those with GA+AA. The effectiveness of the CTLA-4 polymorphisms and haplotype on GVHD after allo-HSCT needs further investigated. The rates of aGVHD III–IV appear lower than our previous report, which reported a rate of 13.4 % [14]. The probable reasons were as followed: (1) the survivors until day 100 of post-allo-HSCT were included in this study; (2) the improvement in GVHD prevention and management over the years as well as the different proportions of donor gender and family relationships in the two reports, [13, 14] with more male donors and more child-to-parent pairs in the current study population (data not shown). Before that published report, since 2009, we intentionally chose young (especially offspring), male donors who were identified to be associated with a lower rate of aGVHD under our protocol.

Perez-Garcia et al. [10] reported that the G genotype of CT60 confers an inferior OS and RFS in 536 HLA-identical sibling donor, but lower incidence of aGVHD. They did not report the impact on cGVHD. Vannucchi et al. [51] reported no impact of +49 and CT60 on OS and RFS in 147 HLA-identical matched unrelated donor transplantation, but identified the worse outcome of CT60 with AA genotype on aGVHD and cGVHD. In an Italian cohort, Azarian et al. [11] did not observe any influence of +49 or CT60 on OS, RFS, or aGVHD in 225 HLA-identical sibling donor, but reported a worse outcome on cGVHD with a G genotype of CT60. Similar findings were reported in a Tunisian cohort [52]. In a recent study of T-depleted allogeneic transplant, the AA genotype of CT60 was associated with an inferior OS and RFS [53]. Mossallam et al. [54] reported that Egyptian recipient with +49 GA/GG allele and lower DFS and OS compared with AA genotype (HR: 2.17, P = 0.03, 95 % CI 1.05–4.48, and HR: 2.54, P = 0.01, 95 % CI 1.16–5.54).

The CTLA-4 polymorphism displays frequencies that are dependent on ethnicity. According to the HapMap Project, the frequencies of the CT60 A allele are 0.460, 0.209 and 0.181 in European, Asians, and sub-Saharan Africans, respectively. Moreover, the frequencies of the +49 A allele are 0.611, 0.637 and 0.314 in European, sub-Saharan Africans, and Asians, respectively. Perez–Garcia et al. [10] reported CT60 genotypes in 536 European donors: GG (24.1 %), GA (47.0 %), and AA (24.1 %). Metaxas et al. [55] reported CT60 genotypes in 79 DLI Europeans donors: GG (45.5 %), GA (32.9 %), and AA (21.5 %). Murase et al. [56] reported 147 Japanese donors with CT60 genotypes: GG (46.9 %), GA (44.9 %), and AA (8.2 %). In our result, 152 Chinese donors had CT60 genotype: GG (63.2 %), GA (34.9 %), and AA (1.9 %). Perez–Garcia et al. [10] reported 536 European donors with +49 genotype, as follows: GG (11.0 %), GA (42.4 %), and AA (46.6 %). Murase et al. [56] reported 147 Japanese donors with +49 genotype: GG (37.4 %), GA (49.7 %), and AA (15.6 %). In our result, 152 Chinese donors had +49 genotype: GG (38.8 %), GA (50.7 %),and AA (10.5 %). Some of the CTLA-4 polymorphisms display frequencies that are different from all the other ethnic groups. The exact effect of the CTLA-4 polymorphism on transplant outcome should be determined in different cohorts with a substantially larger number of subjects.

The SNPs −1661, −318, CT60, and +49 were included in one haplotype block. The SNP −1661 allele A was blocked by the SNP −318 allele C. The haplotype analysis revealed only three haplotypes in the donor population, in this cohort, the frequencies of the haplotype ACGG, ACAA, and GTGA were 64.1, 19.4 and 16.5 %, respectively. All the donors were distributed among the six haplotype combinations. In our study, recipients of donors with the ACGG/ACGG haplotype showed a significantly lower OS in ALL than recipients of donors without the ACGG/ACGG haplotype. Murase et al. [56] reported that the presence of the CTLA-4 CAA (−318, +49, CT60) haplotype reduced the risk of relapse and improved survival in 147 Japanese HLA- identical sibling donors. They reported that three CTLA-4 haplotypes, CTLA-4 (−318, +49, CT60) classified as CGG, CAA and TAG, are present in the Japanese population. Therefore, further investigation is required to elucidate the effect of the CTLA-4 haplotype on the anti-tumor activity of donor-derived T cells.

Our group [57] recently reported selection of the best donor among the related HLA-haplotype-mismatched T-cell replete transplants. Our data suggest that choosing young, male, NIMA-mismatched donors is considerable. Moreover, some potentially important variables such as DSA, CMV serological state, KIR disparity, and other causes may have effect on donor selection. ALL recipients of donors with +49 GG or with haplotype ACGG/ACGG showed significantly lower OS than GA+AA or without ACGG/ACGG. These results hinted that a donor with +49 GG or with haplotype ACGG/ACGG might be inferior to that with GA+AA or without ACGG/ACGG. CTLA-4 polymorphisms and haplotype might be as a potential marker of best donor selection.

The Kaplan–Meier analysis showed a significant lower OS in ALL patients accepting mDLI +49 GG donor or with haplotype ACGG/ACGG than those with AG + AA or without ACGG/ACGG (P = 0.003). Metaxas et al. [55] reported that the presence of a donor CT60 AA or GA genotype vs. a GG genotype was an independent factor for remaining in the complete chimerism/remission post-DLI (OR = 2.61 vs. 0.42, respectively, P = 0.05). These results provided evidence of an independent linkage of the CTLA-4-SNPs and haplotype with an improved chance of sustained CR post-DLI, CTLA-4-SNPs and haplotype may act as a surrogate marker for donor lymphocyte infusion outcome after allo-HSCT for acute leukemia. This means CTLA-4 genotyping might be a substantial diagnostic test before DLI administration.

## Conclusion

In summary, some of the CTLA-4 polymorphisms display frequencies that are different among the different ethnic groups. In our study the CTLA-4 +49 GG and the haplotype (ACGG/ACGG) reduced the overall survival in ALL patients after allo-HSCT from related HLA-haplotype-mismatched donor. Furthermore, knowledge of the CTLA-4 polymorphism and haplotype may provide useful information for donor selection and indications for individual application of immunosuppressive agents and immunotherapy. The exact effect of the CTLA-4 polymorphism and haplotype on transplant outcome should be determined in different cohorts with substantially larger number of subjects.
